# JAC1 suppresses proliferation of breast cancer through the JWA/p38/SMURF1/HER2 signaling

**DOI:** 10.1038/s41420-021-00426-y

**Published:** 2021-04-19

**Authors:** Yanlin Ren, Dongyin Chen, Zurong Zhai, Junjie Chen, Aiping Li, Yan Liang, Jianwei Zhou

**Affiliations:** 1 Department of Molecular Cell Biology & Toxicology, Center for Global Health, School of Public Health, Nanjing Medical University, 211166 Nanjing, China; 2 Jiangsu Key Lab of Cancer Biomarkers, Prevention and Treatment, Collaborative Innovation Center for Cancer Medicine, Nanjing Medical University, 211166 Nanjing, China; 3 Department of Medicinal Chemistry, School of Pharmacy, Nanjing Medical University, 211166 Nanjing, China; 4 Department of Oncology, The First Affiliated Hospital of Nanjing Medical University, 211166 Nanjing, China; 5 Present Address: Nantong Center for Disease Control and Prevention, 226007 Nantong, China

**Keywords:** Breast cancer, Translational research

## Abstract

The overexpression of HER2 is associated with a malignant proliferation of breast cancer. In this study, we developed a non-cytotoxic *JWA* gene activating compound 1 (JAC1) to inhibit the proliferation of HER2-positive breast cancer cells in vitro and in vivo experimental models. JAC1 increased the ubiquitination of HER2 at the K716 site through the E3 ubiquitin ligase SMURF1 which was due to the decreased expression of NEDD4, the E3 ubiquitin ligase of SMURF1. In conclusion, JAC1 suppresses the proliferation of HER2-positive breast cancer cells through the JWA triggered HER2 ubiquitination signaling. JAC1 may serve as a potential therapeutic agent for HER2-positive breast cancer.

## Introduction

Breast cancer poses a huge threat to female health, with the highest morbidity and mortality among female cancers worldwide^1^. Breast cancer will affect as many as one in eight women in high-income countries and remains the leading cancer-related cause of disease burden for women^2,3^. High-income countries represent most of the countries with top incidence rates, whereas low- and middle-income countries (LMICs) represent most of those with the highest mortality rates^4^. The low survival rates in developing countries are interpreted by the scarcity of early detection programs, adequate diagnosis, and treatment facilities, resulting in a high proportion of women presenting with the late-stage disease at diagnosis^5^. Therefore, breast cancer has become a major problem in public health and well concerned in biomedical research^6^. Breast cancer is classified into four subtypes including luminal A, luminal B, HER2-positive, and basal like^7^. Human epidermal growth factor receptor (HER2/ErbB2), a member of the epidermal growth factor receptor (EGFR) family, is negatively or weakly expressed in normal cells. However, it is abundantly expressed in some cases of breast cancer and associated with proliferation and invasion of cancer cells^8,9^. In the past 20 years, breast cancer patients have significantly benefited from monoclonal antibodies targeting HER2 (such as trastuzumab) and tyrosine kinase inhibitors (such as lapatinib)^10–13^. Unfortunately, abnormal activation of the PI3K/AKT/mTOR signaling pathway could promote cancer cells resistant to anti-HER2 therapies and the side effects (e.g., bone marrow suppression and cardiomyopathy) severely restrict the therapeutic efficacy^14–16^. Therefore, the development of the drugs or therapies by appropriately downregulating the overexpressed HER2 (instead of completely blocking HER2) may provide new opportunities for better management of breast cancer cases.

JWA, also known as ARL6IP5, is originally cloned from a retinoic acid-induced Human Bronchial Epithelial cells (HBE cell) differentiation model. The functions of JWA are involved in regulating oxidative stress, DNA repair, cell apoptosis, and differentiation^17,18^. High expression of JWA protein in cancerous tissues is a favorable prognostic indicator for gastric and breast cancer^19,20^. Patients with low expression of JWA in tumor tissues usually suffer worse survival than those with high expression^21,22^. JWA negatively regulates HER2 expression and inhibits cell proliferation and migration in human gastric cancer cells through transcription and ubiquitination-related mechanisms^23,24^. However, it is unclear whether JWA regulates HER2 expression in breast cancer cells.

In the present study, we identified JAC1, an agonist of *JWA* gene, that could down-regulate HER2 expression in breast cancer cells both in vitro and in vivo models. We demonstrated that JAC1 suppressed the proliferative potential via activating JWA expression and then triggering the two-stage ubiquitination modification on HER2.

## Materials and methods

### Cell lines and culture

BT474 and SKBR3 human breast cancer cells were obtained from the American Type Culture Collection (ATCC). The cells were cultured in DMEM supplemented with 20% and 10% fetal bovine serum (FBS), respectively at 37 °C in a humidified atmosphere containing 5% CO_2_. 100 units/mL penicillin and streptomycin (Cellgro, Hemdon, VA) were supplemented in the culture medium. The cell lines were regularly verified by identification tests and were free of mycoplasma contamination. Cycloheximide (CHX) (Sigma-Aldrich, StLouis, MO, USA) and MG132 (Selleck Chemicals, USA) were used at the indicated concentrations.

### Western blotting

Western blot assays were performed based on the previously reported protocols^25^. The following antibodies were used: Anti-β-actin, anti-α-tubulin, anti-GAPDH, anti-HA (1;1000, Beyotime, Jiangsu, China); anti-HER2, anti-HER3, anti-p-p38(Thr180/Thr182); anti-Ub (1:1000, CST, USA); anti-JWA (1:100, Laboratory-made); anti-GATA-1, anti-NEDD4, anti-CBL, anti-SMURF1, anti-ITCH, anti-HER1, and anti-HER4 (1:1000, proteintech, China).

### Colony formation assay

BT474 and SKBR3 cells were seeded into six-well plates with 2 mL DMEM containing 20% and 10% FBS, respectively. Cells were dispersed evenly by slightly shaking the plates. And then BT474 and SKBR3 cells were treated with 1 or 10 μM JAC1 for 10–14 days and not harvested until the formation of visible colonies. Before harvest, the cell colonies were washed twice with PBS, fixed with methanol, and stained with crystal violet. Only the colonies with >50 cells were counted.

### Immunofluorescence microscopy

BT474 and SKBR3 cells were induced with JAC1 or vehicle (DMSO) for 24 h. Subsequently, the cells were fixed with methanol for 30 min, washed with PBST, and 10% normal goat serum for 1 h aiming to block non-specific signals. The cells were incubated with anti-JWA antibody (1:200) and anti-HER2 (1:250) overnight at 4 °C. After washing with PBST, the FITC goat anti-mouse IgG and CY3 goat anti-rabbit IgG (1:100, Beyotime, Jiangsu, China) were used to incubate with cells for 2 h. After washing three times with PBST, the cells were stained with DAPI (Beyotime, Jiangsu, China) for 20 min. The confocal images of the cells were captured using Zeiss AIM software on a Zeiss LSM 700 confocal microscope system (Carl Zeiss Jena, Oberkochen, Germany).

### CRISPR/Cas9 system

The CRISPR/Cas9 system was used to disrupt the expression of the *JWA* gene, as described elsewhere^26^. In brief, a sgRNA sequence was selected using an Optimized CRISPR Design (http://crispr.mit.edu/). The sgRNA sequence for *JWA* was 5′-CCGCGTAGTGAGCAACCTGCTCT-3′. For sequence analysis of the *JWA* gene, the following primer set was used: 5′-CACTCAGTCCCTTGTTCTGTC-3′ (forward) and 5′-GCACCAAGGGCTGAGACTT-3′ (reverse).

### Quantitative real-time PCR assay

Total RNA was extracted from harvested cells using the Trizol reagent (Gibreast cancero, USA) according to the manufacturer’s instructions. Approximately 500 ng of RNA was reversely transcribed with Hiscript Q RT SuperMix for qPCR (Vazyme, Jiangsu, China). And the PCR primers were listed in Supplementary Table 1. Quantitative RT-PCR was carried out with SYBR Green PCR Master Mix (TaKaRa Bio, Japan) using an ABI Prism 7900 Sequence detection system (Applied Biosystems, Canada). The following thermal cycling conditions were used: denaturation at 94 °C for 5 min followed by 36 cycles of denaturation at 94 °C for 35 s, annealing at 56 °C for 30 s and extension at 72 °C for 35 s. The values were calculated as 2^−ddCT^, and the relative fold change was compared to the control groups after being normalized to GAPDH.

### Plasmids and siRNA transfection

The construction of Flag-JWA and Ub plasmids were introduced in our previous study^27^. The HA-HER2 plasmid and corresponding mutants were subcloned into a pcDNA3.1 vector (Generay, Shanghai, China) using HindIII/Xhol sites. The siRNA sequence (5′-CGAGCUAUUUCCUUAUCUC-3′) were used for the *JWA*; the siRNA sequence (5′-GCAGAACAGGCUGAGGAAUTT-3′) were used for the *NEDD4*; the siRNA sequence (5′-CCAGGGAGUGGCUUUACUUTT-3′) were used for the *SMURF1* and a nonsense control siRNA were synthesized by Ribobio (Guangzhou, China). The plasmids and siRNA were transfected into cells with Lipofectamine 3000 according to the manufacturer’s instructions (Invitrogen, Grand Island, NY, USA).

### Immunoprecipitation assay

Cells were washed with PBS twice and incubated with pre-cooled immunoprecipitation (IP) buffer at 4 °C for 30 min. Next, cell lysates were centrifuged at 12,000×*g* for 15 min at 4 °C. The supernatant was immediately transferred into a new centrifugal tube, and then anti-NEDD4 antibody, anti-SMURF1 antibody, anti-HER2 antibody, and appropriate control IgG (mouse, rabbit IgG, corresponding to the host species of the primary antibody) were added in 500 μg total protein and incubated at 4 °C, respectively. After 1 h, the cell lysate was mixed with 20 μL of protein A/G Plus-Agarose (Santa Cruz, USA) at 4 °C overnight. The complex was washed with pre-cooled PBS four times and centrifuged at 4 °C, 1000×*g* for 5 min. Finally, immunoprecipitate was determined by western blot assay.

### Ubiquitination assay

BT474 and SKBR3 cells transiently transfected with Ub for 72 h were incubated with 10 μM JAC1 for 24 h, followed by MG132 (10 μM) induction for another 6 h. The cells were harvested for extracting proteins. Protein samples were incubated with anti-HER2 antibody (1:200, CST, USA) at 4 °C for 1 h, and then added 20 μL Protein A/G Plus-Agarose overnight. A pre-cooled IP buffer was used to wash cells four times. After centrifugation at 4 °C, 1000×*g* for 5 min, the obtained IP product was examined by western blot assay.

### Tissue microarray (TMA) and immunohistochemical (IHC) staining

Human BC tissue microarray (TMA, numbered Brc1802) contained 90 paired cases of cancer tissues and adjacent non-cancerous ones, which was purchased from Shanghai Zhuoli Bio (Shanghai, China). The TMA also provided relevant clinical immunohistochemistry and Fish data of HER2. Tissues samples in the TMA were immune-stained with an anti-JWA monoclonal antibody (1:50). The assessment of the IHC was employed by a semi-quantitative immunoreactivity score (IRS) as previously reported^17,28^. Category A documented the intensity of immunostaining as 0–3 (0, negative; 1, weak; 2, moderate; 3, strong). Category B documented the percentage of immune-reactive cells as 1 (≤25%), 2 (≤50%), 3 (≤75%), and 4 (≤100%). Multiplication of category A and B resulted in an IRS ranging from 0 to 12 for each sample, respectively. The concordance for the IRS of the JWA staining scores between the two pathologists was 82 (91.1%) in 90 cancer cases of the TMA cohort, and a few discrepancies were resolved by consensus using a multi-head microscope. The optimum value of cut-off points of the JWA IRS was 4 in this study. All the samples were classified and analyzed according to these criteria.

### Tumor xenografts

This study was approved by the Ethics Committee of Nanjing Medical University (IACUC-1811067). 5 × 10^6^ BT474 cells suspended in 120 µL of PBS were injected subcutaneously between the shoulder blades of 6-week-old, female severe combined immune deficiency (SCID)–Balb/c mice from Model Animal Research Center of Nanjing University (Nanjing, China). When tumors reached an average size between 100 and 125 mm^3^, the mice were randomly divided into 4 groups, with 9 mice per group. Tumor xenografts were measured with calipers every three days, and calculated using the following formula: Tumor volume = width^2^ × length/2. JAC1 (0, 50, 100 mg/kg) was administered daily by oral gavage in 0.5% hydroxypropyl methylcellulose and 0.1% Tween 80 (Sigma). The animal model was terminated when tumors reached an average size between 2500 and 3000 mm^3^. Half of each tumor tissue was fixed with 4% paraformaldehyde and the other half was frozen at −80 °C for further study. Serum biochemical indicators were tested using an automatic biochemical analyzer (HITACHI, Japan) according to the manufacturer’s operating instructions. Pathological examination of the major organs was performed by routine H&E staining.

### Statistical analysis

The experiments were repeated at least three times. Statistical analysis was performed using SPSS software (version 23.0, Inc., Chicago, IL) and GraphPad Prism 6 software (GraphPad Software, Inc., La Jolla, CA, USA). The data were presented as the means ± SEM. The differences between the two independent groups were analyzed by Student’s *t*-test (unpaired, two-tailed). For each significantly ectopically expressed molecule, Kaplan–Meier overall survival and relapse-free survival analyses were performed using a log-rank test by comparing differences between curves. *P* < 0.05 was considered as statistically significant (*P < 0.05; **P < 0.01; ***P < 0.001).

## Results

### JWA negatively regulates HER2 expression and cell proliferation in breast cancer

**Fig. 1:**
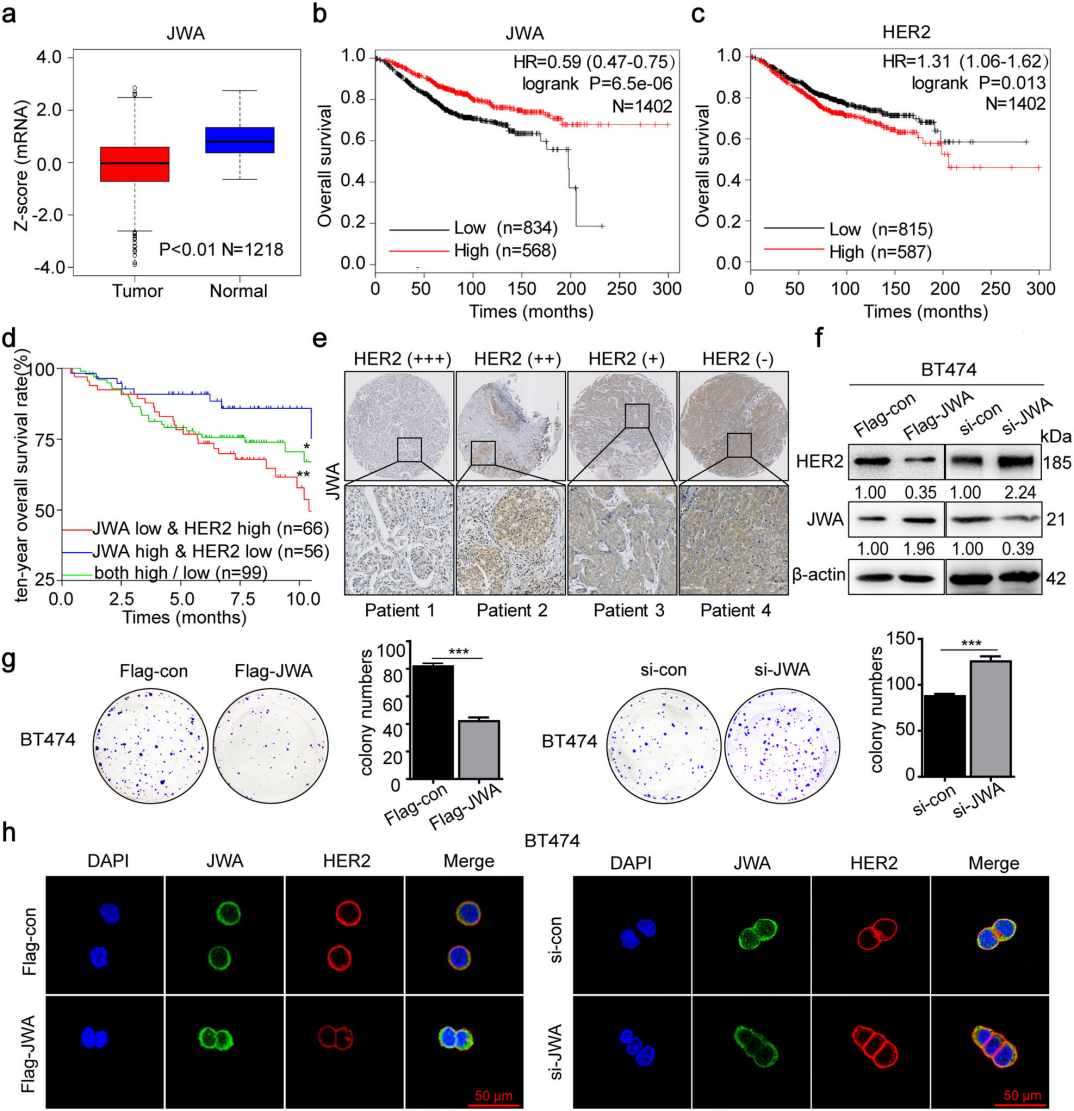
JWA negatively regulates HER2 expression and cell proliferation in breast cancer. **a** The mRNA expressions of *JWA* in breast cancer tissues and adjacent non-cancerous tissues in unpaired cohorts (TCGA database, 1099 cancer samples, 119 non-cancerous samples). **b**, **c** Kaplan–Meier curves depicting OS according to the expression patterns of JWA (**b**) and HER2 (**c**) in the breast cancer cohort. *P* values were calculated with the log-rank test. **d** Kaplan–Meier OS analysis for breast cancer from GEO datasets (GSE88770 and GES42568). **e** Representative images of JWA IHC staining in breast cancer lesions with absent (−), weak (+), moderate (++), strong (+++) expression of HER2 (original magnification, ×2 and ×200). **f** Transfection of either Flag-JWA plasmid or si-JWA and their control into BT474 cells for 72 h, the expressions of HER2 and JWA proteins were determined by western blot. **g** Representative images of the colony formation assay for BT474 after transfection of Flag-JWA, si-JWA or their control. **h** Immunofluorescence imaging of JWA (green), HER2 (red), nucleus labeled as DAPI (blue), the co-localization of the three signals (merge) in BT474 cells transfected with si-JWA (right) or Flag-JWA for 72 h.

To identify the potential biological mechanisms of JWA involved in HER2-positive breast cancer, we firstly compared with normal breast epithelium using data from the interrogated TCGA database and found that JWA was significantly downregulated in breast cancer tissues (*n* = 1218; Fig. 1a) (https://xenabrowser.net/datapages/). Patients with high JWA expression in cancer tissues have longer overall survival (OS) than those with low expression. Patients with high HER2 expression had shorter OS than those with low HER2 expression (*n* = 1402; Fig. 1b, c). To verify these findings, we analyzed survival data from other databases (GSE88770 and GES42568, *n* = 221). The data consistently showed that the patients with low expression of JWA or high expression of HER2 in cancer tissues were unfavorable to the prognosis. In contrast, cases with high expression of JWA and low expression of HER2 in breast cancer tissues had a better outcome (log-rank test, *P < 0.05; **P < 0.01; Fig. 1d). We further determined the protein expressions of JWA and HER2 in human breast cancer tissue microarray (TMA). As predicted, JWA was significantly downregulated in cancer tissues with HER2 overexpression (Fig. 1e). As shown in Supplementary Fig. 1a, b, 32/44 cases showed low expression of JWA in HER2-positive samples. In contrast, 27/46 cases displayed high expression of JWA in HER2-negative ones (*n* = 90; *P* < 0.005).

To determine the potential role of JWA on HER2 expression, Flag-JWA, si-JWA and their controls were constructed and transfected into human breast cancer BT474 cells, respectively. As shown in Fig. 1f, HER2 was downregulated in cells transfected with Flag-JWA; whereas upregulation of HER2 was observed in cells transfected with si-JWA. To determine the regulatory effect of JWA on the proliferation of breast cancer cells, we conducted a colony formation assay. An obvious inhibition of proliferation was observed in BT474 cells transfected with Flag-JWA. On the contrary, increased proliferation was determined in BT474 cells with si-JWA (*P* < 0.005; Fig. 1g). Confocal imaging assay also showed that JWA negatively regulated the intensity of HER2 in BT474 cells (Fig. 1h).

### JAC1 downregulates expression of HER2 and inhibits cell proliferation in breast cancer

**Fig. 2:**
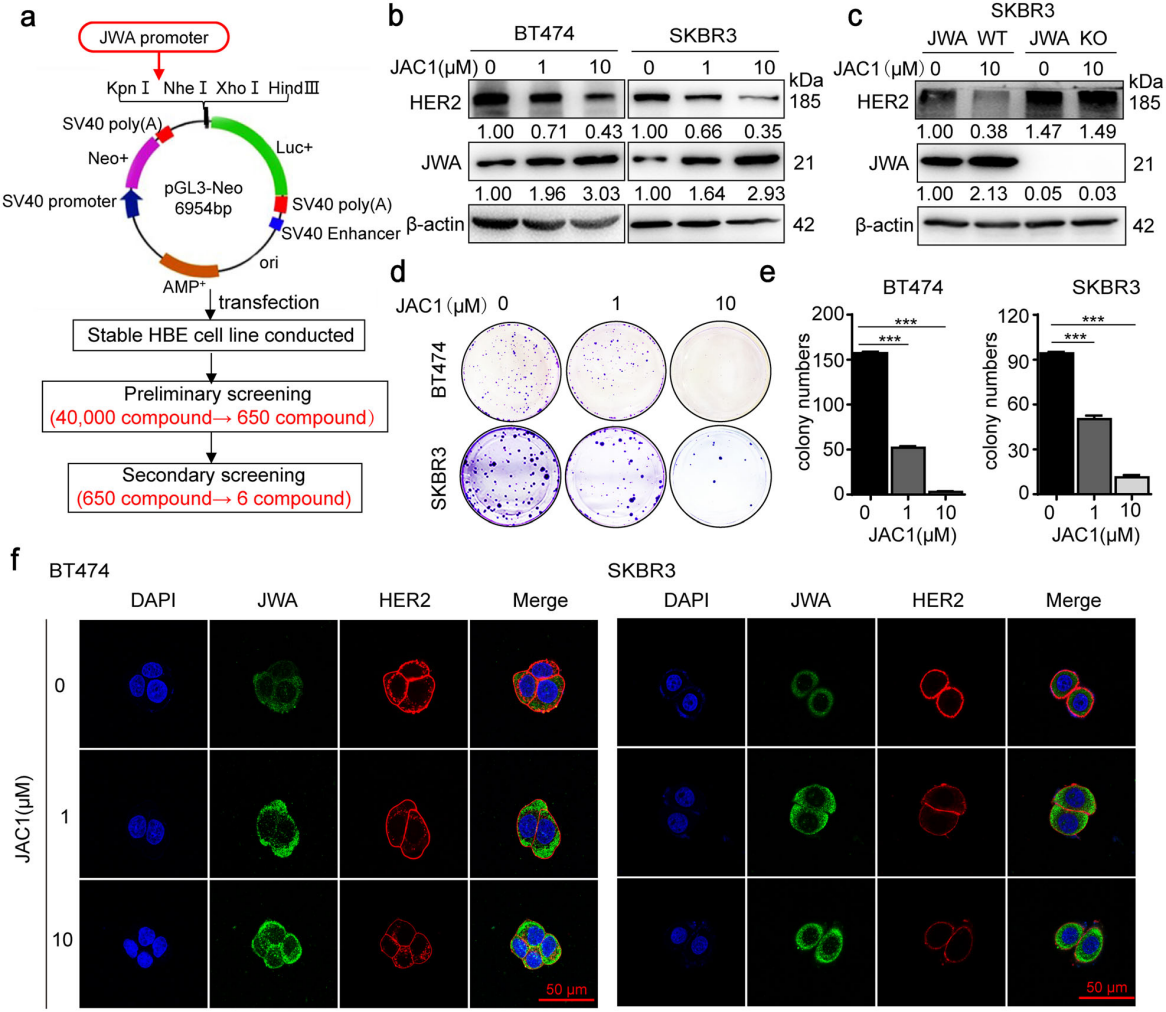
JAC1 downregulates HER2 expression and inhibits cell proliferation in breast cancer. **a** Flow chart of a screening strategy for small molecule compounds *JWA* gene agonist. Through a series of high-throughput screening analyses of 40,000 compounds in the national compound library (Shanghai, China), 650 compounds were obtained. At last, six compounds were identified to have the ability to activate JWA expression according to their molecular weights, lipid water partition coefficients, and chemical structures. **b** Protein expressions of the HER2 and JWA were determined by western blot. **c** JWA/wild-type or JWA/KO cells were treated with JAC1 (10 μM) for 24 h. HER2 and JWA protein expressions were determined by western blot. **d**, **e** Representative images of the colony formation assay for BT474 and SKBR3 cells after treatment with different concentrations of JAC1 (0, 1, 10 μM). **f** BT474 and SKBR3 cells were treated with JAC1 (0, 1, 10 μM) for 24 h, immunofluorescence imaging showed expressions of JWA (green), HER2 (red), nucleus (blue), the co-localization of the three signals (merge).

To find the potent agonist of *JWA* gene, we constructed JWA promoter-containing reporter gene plasmid and stably transfected it into HBE cells. A series of high-throughput screening assays on 40,000 compounds were conducted in the national compound library, Shanghai Institute of Materia Medica, Chinese Academy of Sciences (Shanghai, China). And 650 compounds were obtained for further investigation. Finally, based on the molecular weights, lipid water partition coefficients, and chemical structures of the compounds, six compounds (JAC1~JAC6) were identified to have the ability to activate JWA expression (Fig. 2a). As shown in Supplementary Fig. 2a, b, JAC1 indicated the best effect on inhibiting breast cancer cell proliferation compared to the others. In addition, JAC1 displayed better capacities in activating JWA and downregulating HER2 in BT474 cells than others (Supplementary Fig. 2c). The chemical structure formula of JAC1 was shown in Supplementary Fig. 3a. The effects of JAC1 on the expressions of JWA and HER2 were dose-dependent in both BT474 and SKBR3 cells (Fig. 2b). The IC_50_ values of JAC1 were between 25 and 50 μM in BT474 and SKBR3 cells (Supplementary Fig. 3a, b).

To confirm whether JWA was necessary for JAC1 to induce the downregulation of HER2 in breast cancer cells, we constructed JWA knockout (KO) SKBR3 cells by CRISPR/Cas9 technique. DNA sequencing results showed that the 313th base (A) of *JWA* DNA sequence was replaced by CT bases (Supplementary Fig. 3d). An off-target assay of JAC1 on HER2 expression was also carried out in JWA KO SKBR3 cells. As indicated in Fig. 2c, JAC1 activated JWA and significantly suppressed HER2 expression in JWA wild-type (WT) SKBR3 cells; however, JAC1 was unable to down-regulate HER2 expression in JWA KO SKBR3 cells. Colony formation assay showed that JAC1 inhibited the proliferation of BT474 and SKBR3 cells in a dose-dependent manner (Fig. 2d, e). The dose-dependent induction of JWA intensity, as well as an inhibitory effect of HER2 intensity, were observed by confocal imagining. JAC1 treatment showed no effect on HER2 localization in both BT474 and SKBR3 cells (Fig. 2f).

### JAC1 downregulates expression of HER2 via the ubiquitin–proteasome pathway

**Fig. 3:**
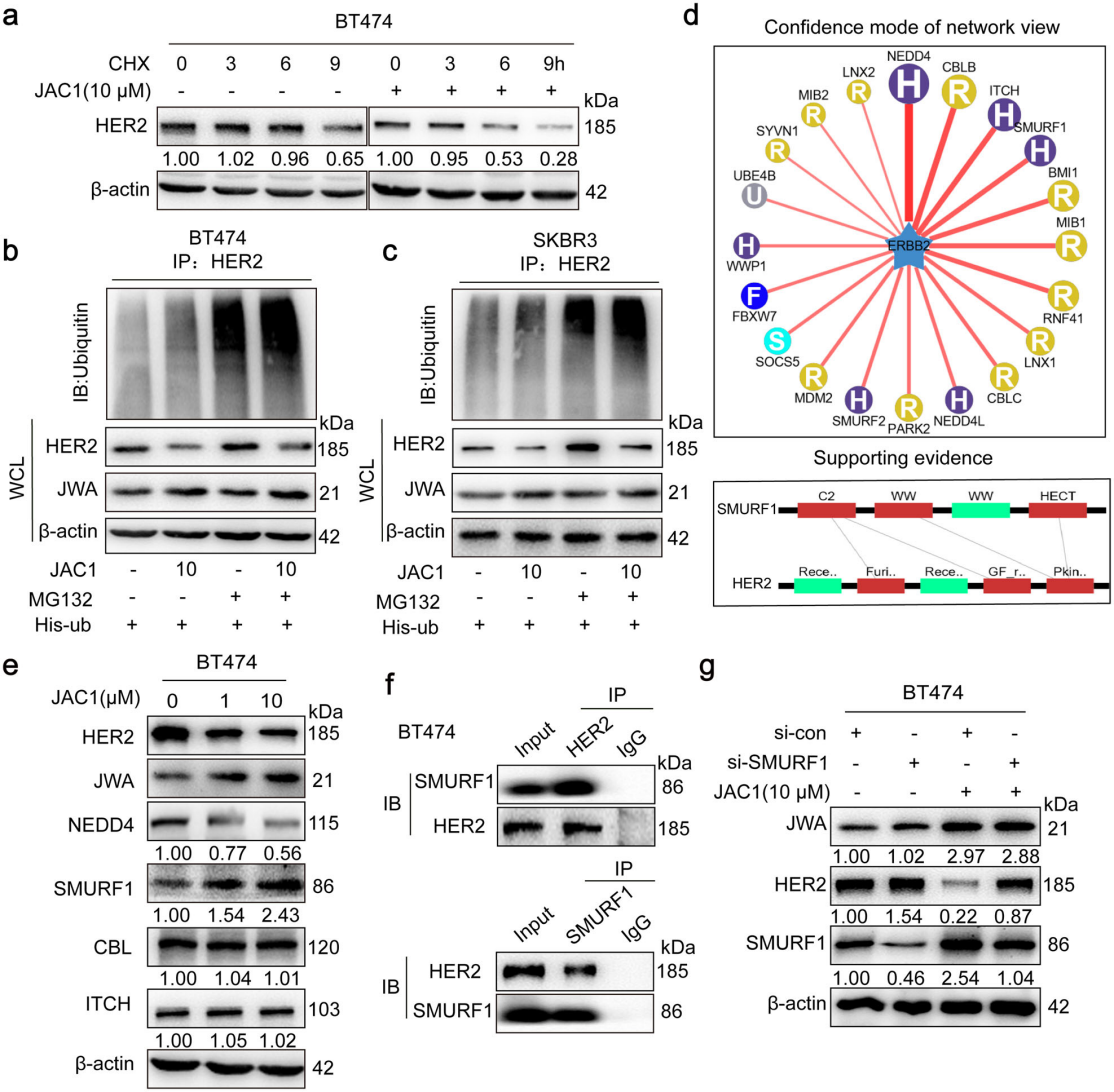
JAC1 downregulates HER2 expression via ubiquitin–proteasome pathway. **a** BT474 cells were treated with the JAC1 (0, 1, 10 μM) for 24 h, followed by Cycloheximide (CHX, 100 μg/mL) for 0, 3, 6, 9 h. HER2 protein expressions were determined by western blot. **b**, **c** Ubiquitination of HER2 was induced by JAC1. Ubiquitination of the HER2 protein was immunoprecipitated and detected a ubiquitin antibody. **d** The HER2-targeting E3 ubiquitin ligases were predicted using the public database website (ubibrowser.ncpsb.org/). **e** After BT474 cells were treated with the JAC1 (0, 1, 10 μM) for 24 h, the expressions of E3 ligases which were ranked at the top were determined by western blot. **f** BT474 cells were pretreated with MG132 (10 μM) for 6 h, and the endogenous protein–protein interaction between SMURF1 and HER2 was assessed by IP and followed by western blot. **g** Western blotting analysis of HER2 and SMURF1 in BT474 cells treated with JAC1 or si-SMURF1 and corresponding controls.

To determine how JAC1 downregulates HER2 in breast cancer cells, we examined mRNA expression of *HER2* by RT-PCR. The data showed that JAC1 increased *JWA*, but had no effect on *HER2* at mRNA expressions in both BT474 and SKBR3 cells (Supplementary Fig. 4a, b). At protein expression, HER2 degradation was obviously accelerated by JAC1 treatment in both BT474 and SKBR3 cells at the indicated time points (Fig. 3a and Supplementary Fig. 4c). We further demonstrated that ubiquitin-modified HER2 was obviously upregulated in both BT474 and SKBR3 cells after JAC1 exposure (Fig. 3b, c). The potential E3 ubiquitination ligase of HER2 was subsequently searched in the UbiBrowser (ubibrowser.ncpsb.org/)^29^. The data indicated that NEDD4, CBL, ITCH, and SMURF1 may be involved in ubiquitin-mediated degradation of HER2 triggered by JAC1 (Fig. 3d). As a result, among the four candidates, only SMURF1 expression was upregulated but NEDD4 was downregulated by JAC1 treatment in both BT474 and SKBR3 cells (Fig. 3e and Supplementary Fig. 4d). To confirm this, *JWA* transfection assay was completed. Similar results were obtained after transfection of either Flag-JWA or si-JWA in SKBR3 cells (Supplementary Fig. 4e). These data suggested that SMURF1 may be the E3 ubiquitin ligase for HER2. We then conducted co-immunoprecipitation experiments to confirm the potential interaction between SMURF1 and HER2. As predicted, an interaction between HER2 and SMURF1 was identified in BT474 and SKBR3 cells (Fig. 3f and Supplementary Fig. 4f). Unfortunately, there was no interaction between HER2 and NEDD4, HER2 and CBL or HER2 and ITCH (Supplementary Fig. 4g–l). Furthermore, the inhibition of SMURF1 expression by si-SMURF1 could reverse the degradation of HER2 by JAC1 treatment (Fig. 3g).

### K716 of HER2 is the ubiquitination target by SMURF1

**Fig. 4:**
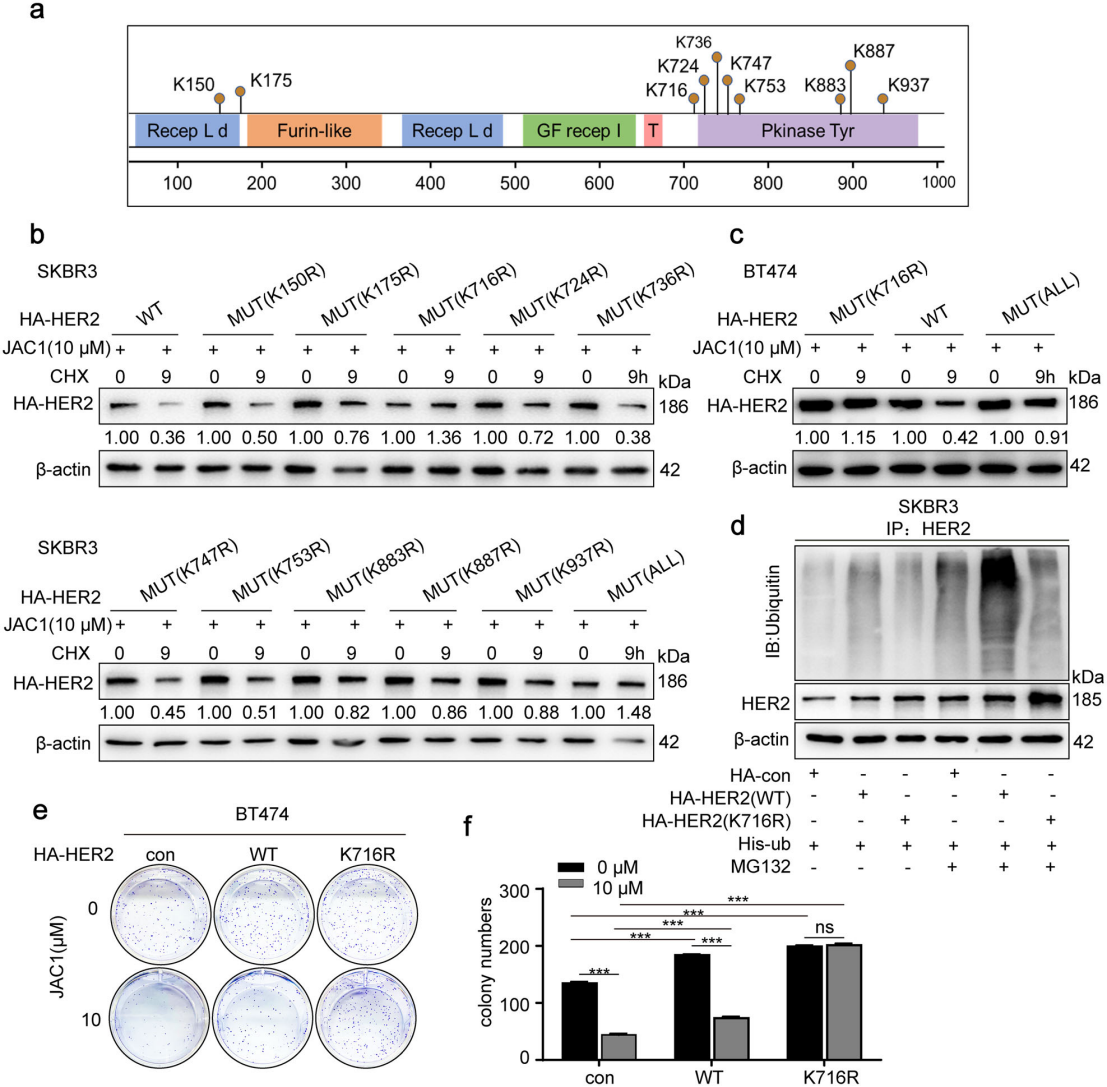
K716 of HER2 is the ubiquitination target by SMURF1. **a** Data from the PhosphoSitePlus (https://www.phosphosite.org) showed the potential sites required for ubiquitination of HER2. **b** SKBR3 cells were transfected with His-HER2 (WT) or 11 mutants (10 separate single amino acid mutants and all 10 mutated amino acids in HER2), treated with the 10 μM JAC1 for 24 h, followed by exposure to CHX (100 μg/mL) for 9 h. Protein expression of HER2 was detected by western blot. **c** BT474 cells were transfected with HA-HER2 (WT), HA-HER2 (K716) or HA-HER2 (ALL) for 72 h, treated with the 10 μM JAC1 for 24 h, followed by exposure to 100 μg/mL of CHX for 9 h; the protein expression of HER2 was determined by western blot. **d** SKBR3 cells were co-transfected with His-Ub, HA-HER2 (WT), or HA-HER2 (K716) for 72 h, followed by pretreatment with MG132 (10 μM) for 6 h. Ubiquitinated HA-HER2 was determined by IP followed immunoblot. **e** Colony formation assay was completed for WT/K716R HER2 transfected BT474 cells and followed by JAC1 (10 μM) treatment. **f** The quantitative data of colony numbers.

To identify the potential target amino acids in HER2 by SMURF1 ubiquitination, we searched an online database (http://www.phosphosite.org/). As shown in Fig. 4a, there were 10 candidate sites in the amino acid sequence of the HER2 protein. We then constructed all or separated 10 amino acids mutants in the HER2 sequence and replaced lysine (K) with arginine (R), respectively. Next, wild or mutant HER2 plasmids were transfected into SKBR3 cells respectively, followed by JAC1 treatment. As a result, only HER2 (K716R) and HER2 (ALL) (all 10 sites mutant) were resistant to degradation of HER2 (Fig. 4b). To confirm this finding obtained from SKBR3 cells, HER2 (K716R), HER2 (WT), and HER2 (ALL) mutants were transfected into BT474 cells, respectively, followed by treatment of JAC1. Similar results were obtained that JAC1 only accelerated degradation of HER2 (WT), rather than HER2 (K716R) and HER2 (mutant ALL) (Fig. 4c). Then, the ubiquitination of HA-HER2 was attenuated by MG132 treatment in HER2 (K716R) SKBR3 cells compared to those with HER2 (WT) cells (Fig. 4d). JAC1 (10 μM) treatment obviously suppressed cell proliferation in both HER2 (WT) and non-transfected control BT474 cells, but it had no effect on HER2 (K716R) BT474 cells (Fig. 4e–f). Thus, the effect of JAC1 on the proliferation of breast cancer cells depended on the presence of amino acid K716 in HER2.

### JAC1 modulates SMURF1 through JWA-p38-GATA-1-NEDD4 axis

**Fig. 5:**
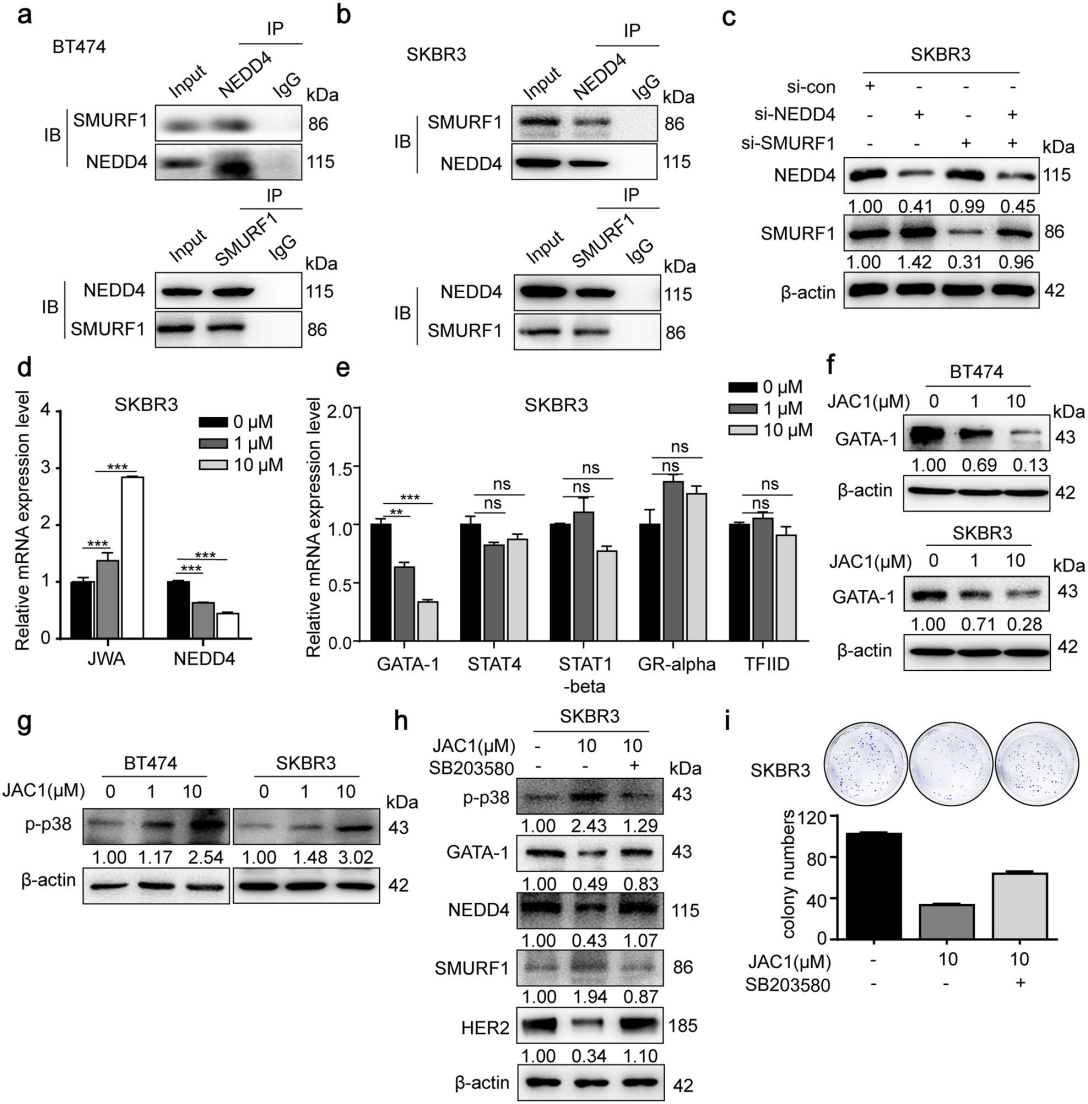
JAC1 modulates SMURF1 through JWA-p38-GATA-1-NEDD4 axis. **a**, **b** The endogenous protein–protein interaction between NEDD4 and SMURF1 was identified by IP and western blot in both BT474 and SKBR3 cells. **c** Western blot analysis of NEDD4 and SMURF1 of SKBR3 cells transfected with si-NEDD4 or si-SMURF1. **d** The mRNA expressions of *NEDD4* and *JWA* were detected by real-time PCR in SKBR3. The values were calculated as 2^−ddCT^, and the relative fold change was compared to the control groups after being normalized to GAPDH. **e** The mRNA expressions of *GATA-1*, *STAT4*, *STAT1-beta*, *GR-alpha*, and *TFIID* were detected by real-time PCR in SKBR3. The values were calculated as 2^−^^ddCT^, and the relative fold change was compared to the control groups after being normalized to GAPDH. **f** The regulations of JAC1 on GATA-1 were determined in both BT474 and SKBR3 cells. **g** JAC1-induced activation of p-p38 was determined by western blot in both BT474 and SKBR3 cells. **h** Western blot analysis of p-p38, GATA-1, NEDD4, SMURF1, and HER2 of SKBR3 cells treated with JAC1 or SB203580. **i** The inhibition of JAC1 on colony formation via the p38 MAPK pathway was determined in SKBR3 cells.

Given that JAC1 was a potent activator of SMURF1, we desired to identify how JAC1 upregulates SMURF1. As predicted, NEDD4 was the most crucial E3 ubiquitin ligase of SMURF1 (Supplementary Fig. 5a) since it was dose-dependently downregulated by JAC1 treatment (Fig. 3e and Supplementary Fig. 4d). The expression patterns of SMURF1 and NEDD4 were quite opposite after JAC1 exposure. In addition, co-IP experiments manifested the interaction between SMURF1 and NEDD4 (Fig. 5a, b). However, the interaction between SMURF1 and NEDD4L was not identified (Supplementary Fig. 5b). When SKBR3 cells were transfected with si-NEDD4, the expression of NEDD4 was decreased while the expression of SMURF1 was increased significantly. However, no change of NEDD4 expression was observed after transfection of si-SMURF1 in SKBR3 cells. These results indicated that NEDD4 was an upstream negative regulatory molecule of SMURF1 (Fig. 5c).

To elucidate how JAC1 downregulates NEDD4 expression, we determined mRNA expression of *NEDD4* by RT-PCR. The data showed that JAC1 dose-dependently downregulated mRNA expression of *NEDD4* (Fig. 5d and Supplementary Fig. 5c). Further, online prediction (UCSC and ALGGEN-PROMO) indicated that several transcription factors (GATA-1, ER-alpha, STAT4, STAT1-beta, GR-alpha, and TFIID) may be involved in JAC1-mediated NEDD4 downregulation (Supplementary Fig. 5d). RT-PCR results showed that JAC1 downregulated mRNA expression of *GATA-1*, but had no effect on the mRNA expression of *STAT4*, *STAT1-beta*, *GR-alpha,* and *TFIID* (Fig. 5e). Western Blot results showed that JAC1 treatment induced a dose-dependent increase of JWA and a decrease of GATA-1 expression (Fig. 5f). Recent studies have revealed that JWA inhibits cell migration by activating the MAPK signaling pathway^18,30^, and TNF-α represses GATA-1 through the activation of p38 MAPK signaling^31^. Here we identified that expression of GATA-1 was negatively regulated by p38 MAPK signaling, which was mediated by JAC1 (Fig. 5g). To confirm this speculation, p38 inhibitor SB203580 was used to block p38. Data showed that the expression of p-p38 induced by JAC1 was mostly prevented by SB203580. Furthermore, the inhibitory effects of JAC1 on the expressions of GATA-1, NEDD4, and HER2 were mostly reversed by SB203580 treatment; meanwhile, the positive effect of JAC1 on the expression of SMURF1 was also suppressed by SB203580 in SKBR3 cells (Fig. 5h). To further confirm the involvement of this pathway in JAC1-regulated proliferation in breast cancer, a colony formation assay was conducted. The data showed that the inhibition of JAC1 on the proliferation of SKBR3 cells was partly reversed by SB203580 (Fig. 5i). Taken together, we have revealed that JAC1 inhibited the proliferation of breast cancer cells by activating SMURF1.

### JAC1 suppresses tumor growth in breast cancer xenografted mice

**Fig. 6:**
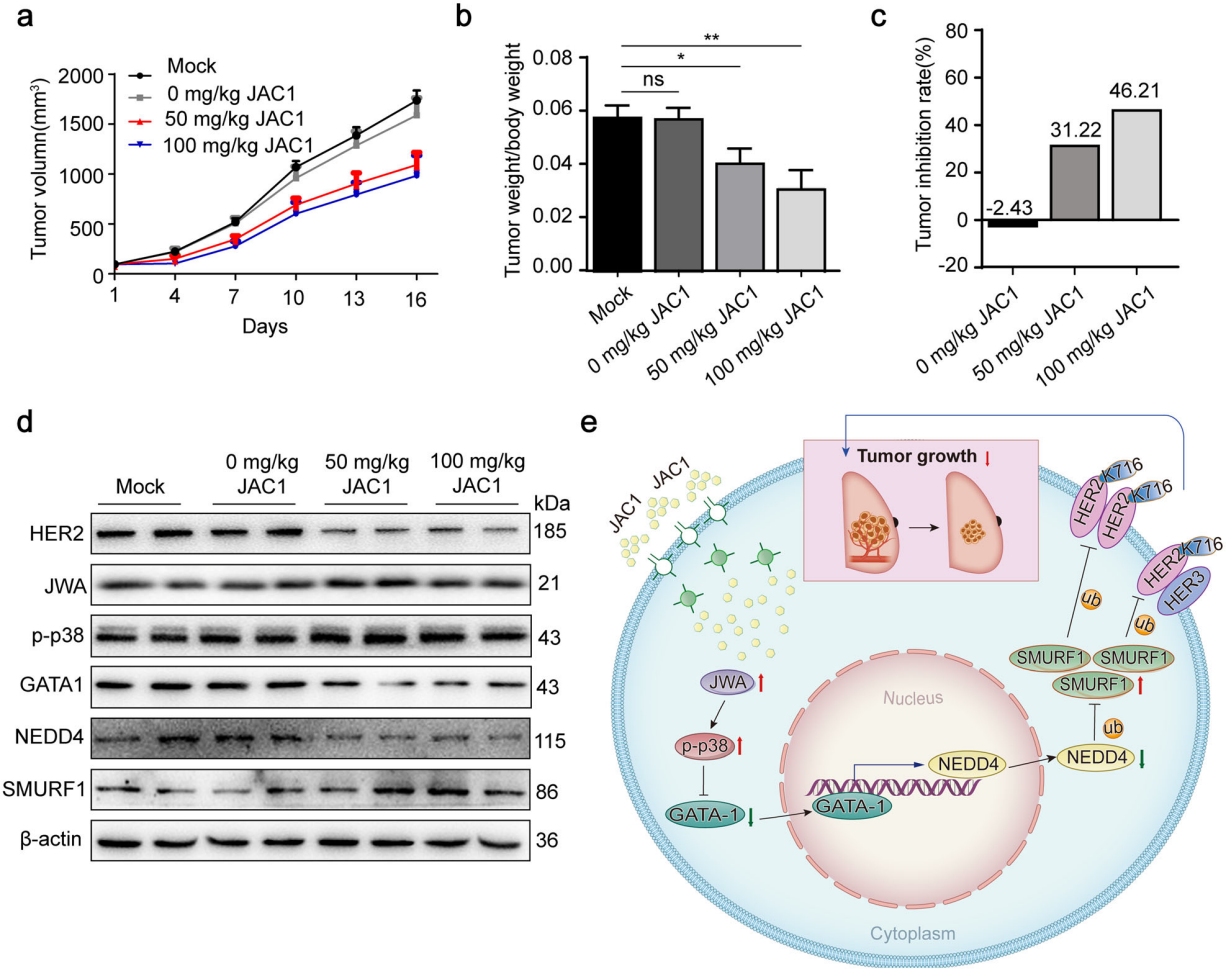
JAC1 modulates HER2 ubiquitination and inhibits tumor growth in vivo. **a** The curve of tumor growth of BT474 xenografted breast cancer after JAC1 treatment. **b**, **c** The ratio of tumor weight/body weight in each group (**b**), tumor inhibition rate by JAC1 (**c**). **d** The expressions of cell proliferation biomarkers and mechanism biomarkers in isolated representative tumor tissues of each group; all the biomarkers were determined by western blot. **e** A schematic overview of how JAC1 degrades HER2 in breast cancer cells. *P < 0.05; **P < 0.01; ***P < 0.001. N.S. no significant differences.

To evaluate the translational significance of JAC1, we established a mouse xenograft model of breast cancer BT474 cells in vivo. As shown in Fig. 6a, JAC1 dose-dependently inhibited the growth of xenografted tumors in mice. A similar trends of tumor weight/body weight were shown in Fig. 6b. The tumor inhibition rate by JAC1 alone was 31.22% and 46.21% in 50 mg/kg and 100 mg/kg groups, respectively (Fig. 6c). In addition, compared to the mock group, the solvent showed no significant effect on tumor growth (Fig. 6a–c and Supplementary Fig. 6a, b). The mechanism of inhibiting breast cancer by JAC1 in vivo was also determined. As shown in Fig. 6d, expression of HER2 was dose-dependently reduced by JAC1 treatment. Importantly, the mechanistic biomarkers including JWA, p-p38, GATA-1, NEDD4, and SMURF1 were correspondingly changed in tumor tissues. H&E staining revealed that JAC1 treatment did not show obvious injuries in mice organs, including lung, liver, spleen, kidney, and myocardium (Supplementary Fig. 6c). The serum biochemical parameters indicated a favorable improvement in antioxidant (SOD), liver function (ALT/AST), myocardial enzymes (CKMB/CK), and lipid metabolisms (TG) after JAC1 treatment (Table 1). The mechanism of JAC1 in the inhibition of breast cancer cell proliferation has been summarized in Fig. 6e.

**Table 1:** The serum biochemical indexes (21 parameters) in control and 100 mg/kg JAC1-treated mice.

Analysis	Groups		
	Mock	0 mg/kg JAC1	100 mg/kg JAC1
[ALT]	29.33 ± 2.52	30 ± 4.24	23.75 ± 2.22*
[AST]	134.75 ± 18.46	136 ± 16.19	97 ± 19.61*
[TP]	39.03 ± 3.44	42.24 ± 5.07	42.16 ± 5.21
[ALB]	26.23 ± 0.81	27.35 ± 3.48	29.95 ± 1.18**
[TBIL]	0.47 ± 0.15	0.43 ± 0.15	0.58 ± 0.1
[ALP]	212.25 ± 41.05	235.75 ± 68.86	186.8 ± 33.89
[GGT]	4.67 ± 1.15	4.01 ± 0.01	4.22 ± 1.41
[GLU]	8.6 ± 2.27	8.53 ± 3.87	11.05 ± 4.03
[BUN]	8.93 ± 2.4	10.1 ± 1.42	12.2 ± 1.83
[CREA]	19.33 ± 1.15	19.5 ± 1.29	20.75 ± 0.96
[UA]	204.67 ± 52.6	187.33 ± 23.97	169.5 ± 24.12
[Ca]	2.44 ± 0.17	2.4 ± 0.12	2.39 ± 0.11
[P]	2.72 ± 0.65	2.9 ± 0.34	2.5 ± 0.37
[CHOl]	2.58 ± 0.17	2.48 ± 0.19	2.85 ± 0.48
[TG]	1.25 ± 0.37	1.17 ± 0.32	0.63 ± 0.12*
[HDLC]	1.48 ± 0.23	1.65 ± 0.18	1.69 ± 0.24
[LDLC]	0.46 ± 0.04	0.48 ± 0.04	0.44 ± 0.07
[LDH]	1777.25 ± 519.45	2010.25 ± 308.79	1344.83 ± 490.88
[CKMB]	437 ± 127.14	513.25 ± 26.99	296.17 ± 48.52*
[SOD]	51 ± 18.19	70.5 ± 14.62	92.17 ± 15.3**
[CK]	845.5 ± 135	948 ± 163.81	478.2 ± 82.87***

Data were presented as mean ± standard deviation (*n* = 4).

*P < 0.05.

**P < 0.01.

***P < 0.001.

HER2 is known as a member of the EGFR family, and the K716 site was identified as a homologous amino acid in the protein sequence of all four EGFR members (Supplementary Fig. 7a). We then determined the expression of HER1, HER3, and HER4 in JAC1-treated SKBR3 cells. As predicted, JAC1 dose-dependently suppressed expressions of all these EGFR members (Supplementary Fig. 7b).

## Discussion

Ubiquitination is one of the mechanisms by which cells maintain homeostasis of protein expression levels. Overexpression of HER2 in some cancers, such as breast cancer, may be caused by failure or insufficiency of ubiquitination. Although previous studies have reported the regulatory role of E3 ubiquitin in breast cancer cells, the relationship between the inactivation of HER2 E3 ubiquitin ligases and breast cancer progression has not been investigated. In the present study, we identified for the first time that JAC1, the *JWA* gene agonist, could inhibit the proliferation of breast cancer cells by enhancing the ubiquitin modification of HER2 at the K716 site and accelerating its degradation through SMURF1 E3 ligase. The in vivo data showed that JAC1 dose-dependently suppresses proliferation of HER2-positive breast cancer without toxic side effects in the experimental mouse. It is suggested that JAC1 has potential in drug development and may be utilized as a fundamental therapy for HER2-positive breast cancer.

*JWA* gene is biologically conserved in evolution and involves several essential functions, including regulations of cell differentiation, DNA repair, cytoskeleton organization, and maintenance of the homeostasis of glucose and lipid metabolism^23,32,33^. The biological functions of the JWA are achieved largely by regulating the MAPK signaling pathway, which is essential for maintaining basic life activities in organisms^34^.

HER2 is known as a member of the EGFR family, which contains four members, including EGFR (HER1), HER2 (ErbB2), HER3, and HER4^35^. HER2-positive breast cancer cells usually contain homo- or heterodimers of EGFR members, such as HER2/HER2 or HER2/HER3^36–38^. In the present study, we identified that K716 is the common amino acid in all four EGFR family members, and JAC1 downregulated expressions of HER1, HER3, and HER4 in breast cancer cells.

The current HER2-targeted therapies (trastuzumab, pertuzumab, and lapatinib) for clinical treatment of HER2-positive breast cancer have largely improved the prognosis of patients^39^. Unfortunately, the therapies are always accompanied by inevitable side effects and drug resistance, which have become a challenge in clinical application^40,41^. Although the antibody-drug conjugates (ADC) drugs have shown powerful therapeutic effects than HER2-target antibody alone^42,43^, their side effects have become the bottleneck and need to be concerned. In addition, none of the above drugs or therapies can degrade overexpressed HER2 or HER3, which may inevitably contribute to the development of drug resistance and side effects. Therefore, the urgent need for treatment of HER2-positive breast cancer is to develop novel drugs or therapies to degrade the overexpressed HER2 in cancer cells, thereby reversing HER2-mediated excessive proliferation and metastasis in several cancers.

In this study, JAC1 was identified to degrade the overexpressed HER2 and other EGFR members in breast cancer cells. This may provide new insight into the clinical therapy for breast cancer with overexpressed EGFR. EGFR family mutations or rearrangements lead to drug resistance, which is the current challenge for targeted drugs. Fortunately, some mutations in the *EGFR* gene in lung cancer cells bring patients the opportunity to receive targeted therapy and improve their prognosis. However, further studies are needed to clarify whether JAC1 is also effective against HER2 or EGFR mutations, as well as the drug resistance of related cancer cells. NEDD4 is associated with breast cancer progression and predicts a poor prognosis. Upregulation of NEDD4 mediates cell migration in lung cancer cells, while downregulation of NEDD4 inhibits cell growth and invasion, and induces cell apoptosis in bladder cancer cells^44–46^. These evidences support that the anticancer effect of JAC1 may be associated with its indirect inhibition on NEDD4.

We acknowledge that the clues found in this study are very preliminary because epidermal growth factor receptors such as HER2 are often allosteric as hetero/homodimers adaptations to targeted drug therapy. Future research will focus on the regulatory role of ubiquitin-related mechanisms in cancers that have developed resistance to HER2-targeted therapy.

In conclusion, JAC1 was identified as an indirect inhibitor of HER2. Its mechanism of action in breast cancer was through the cascade regulations of the two E3 ubiquitin ligases (NEDD4 and SMURF1), which eventually enhanced the ubiquitination modification of HER2 at K716. JAC1 may have equal significance in EGFR positive cancer therapy.

## Supplementary information

Supplementary Table 1

Supplementary Figure 1

Supplementary Figure 2

Supplementary Figure 3

Supplementary Figure 4

Supplementary Figure 5

Supplementary Figure 6

Supplementary Figure 7
