# Supplementary material for: JAC1 suppresses proliferation of breast cancer through the JWA/p38/SMURF1/HER2 signaling
Source: Cell Death Discov. 2021 Apr 19;7:85. doi: 10.1038/s41420-021-00426-y (PMC8055679; doi:10.1038/s41420-021-00426-y)
Supplement: Supplementary file 1 — Supplementary Table 1 [file 41420_2021_426_MOESM1_ESM.docx]

| Gene | Forward primer (5’- 3’) | Reverse primer (5’- 3’) |
| --- | --- | --- |
| HER2 | TGTGACTGCCTGTCCCTACAA | CCAGACCATAGCACACTCGG |
| JWA | GGAGGAGTCATTGTGGTGC | GAAGTCTCAGGGATGCGTG |
| NEDD4 | TCAGGACAACCTAACAGATGCT | TTCTGCAAGATGAGTTGGAACAT |
| GATA-1 | CTGTCCCCAATAGTGCTTATGG | GAATAGGCTGCTGAATTGAGGG |
| STAT4 | TGTTGGCCCAATGGATTGAAA | GGAAACACGACCTAACTGTTCAT |
| STAT1-beta | CAGCTTGACTCAAAATTCCTGGA | TGAAGATTACGCTTGCTTTTCCT |
| GR-alpha | ACAGCATCCCTTTCTCAACAG | AGATCCTTGGCACCTATTCCAAT |
| TFIID  GAPDH | CCACTCACAGACTCTCACAAC  GCCGGTGCTGAGTATGTC | CTGCGGTACAATCCCAGAACT  CTTCTGGGTGGCAGTGAT |

**Table S1. qRT-PCR primers**
