# Supplementary figures and images for: JAC1 suppresses proliferation of breast cancer through the JWA/p38/SMURF1/HER2 signaling
Source: Cell Death Discov. 2021 Apr 19;7:85. doi: 10.1038/s41420-021-00426-y (PMC8055679; doi:10.1038/s41420-021-00426-y)

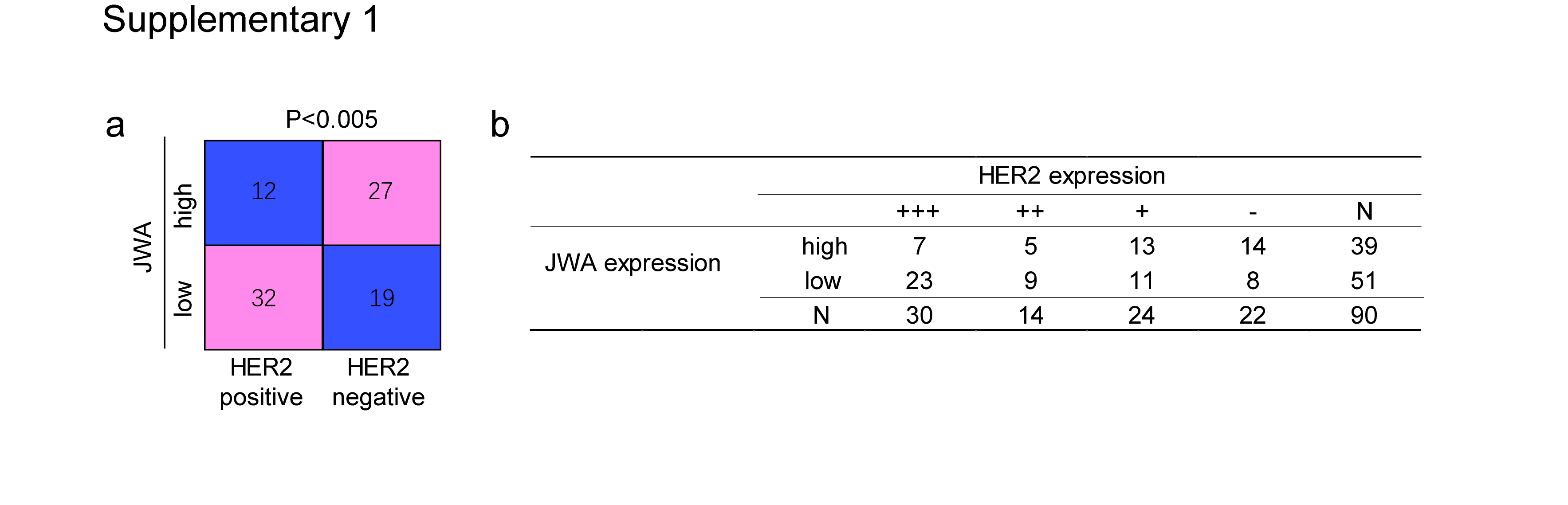

Supplement: Supplementary file 2 — Supplementary Figure 1 [file 41420_2021_426_MOESM2_ESM.tif]

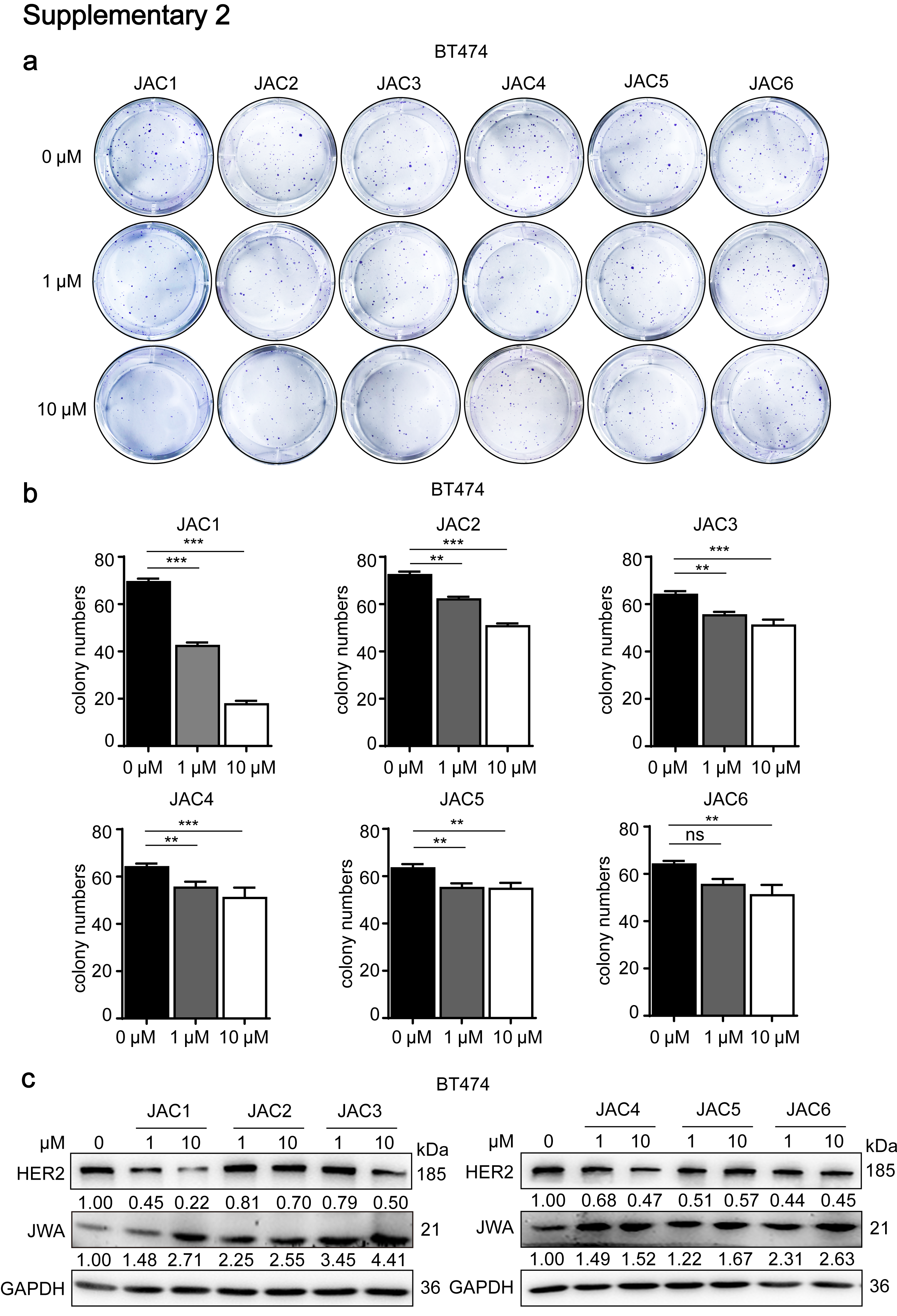

Supplement: Supplementary file 3 — Supplementary Figure 2 [file 41420_2021_426_MOESM3_ESM.tif]

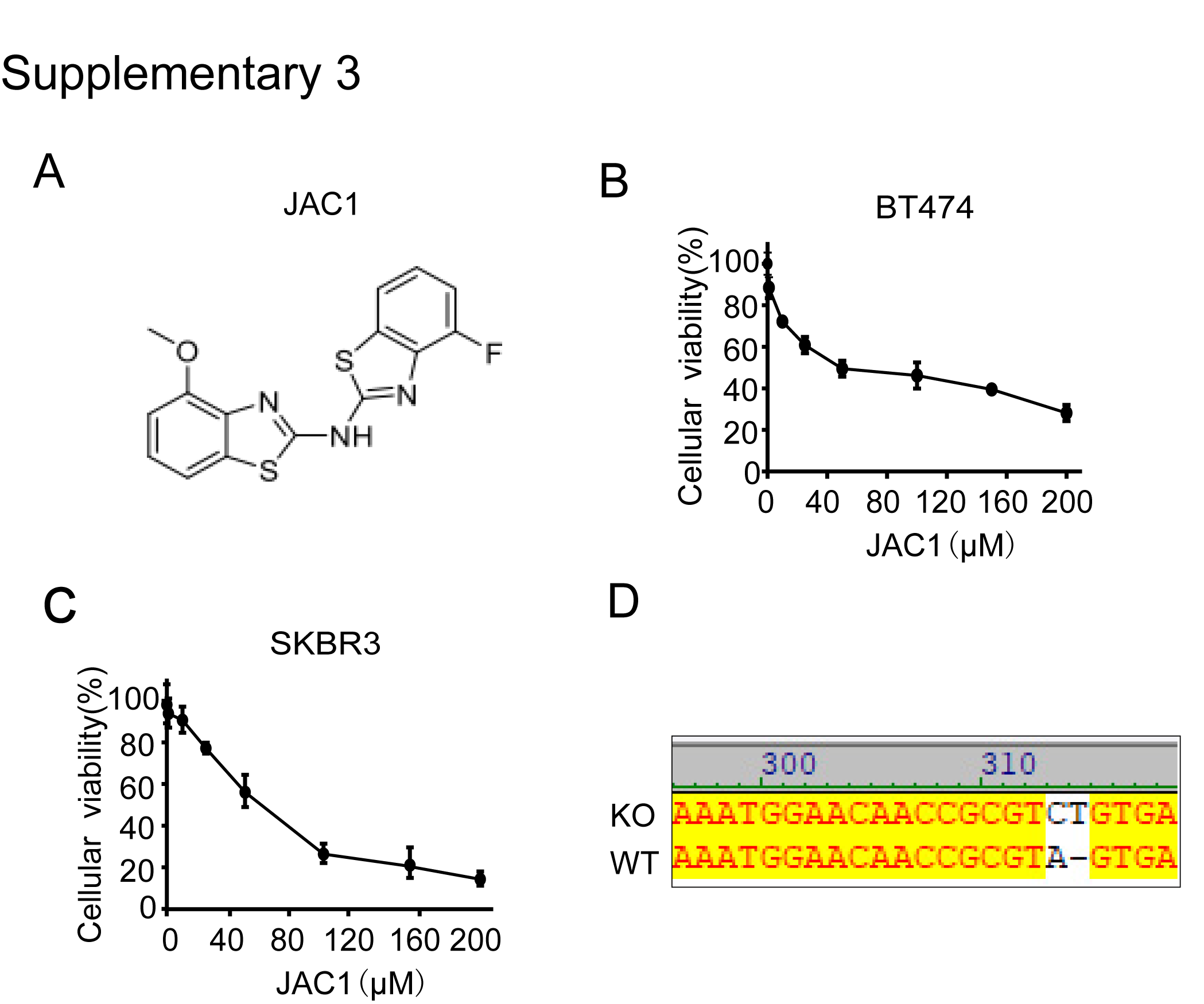

Supplement: Supplementary file 4 — Supplementary Figure 3 [file 41420_2021_426_MOESM4_ESM.tif]

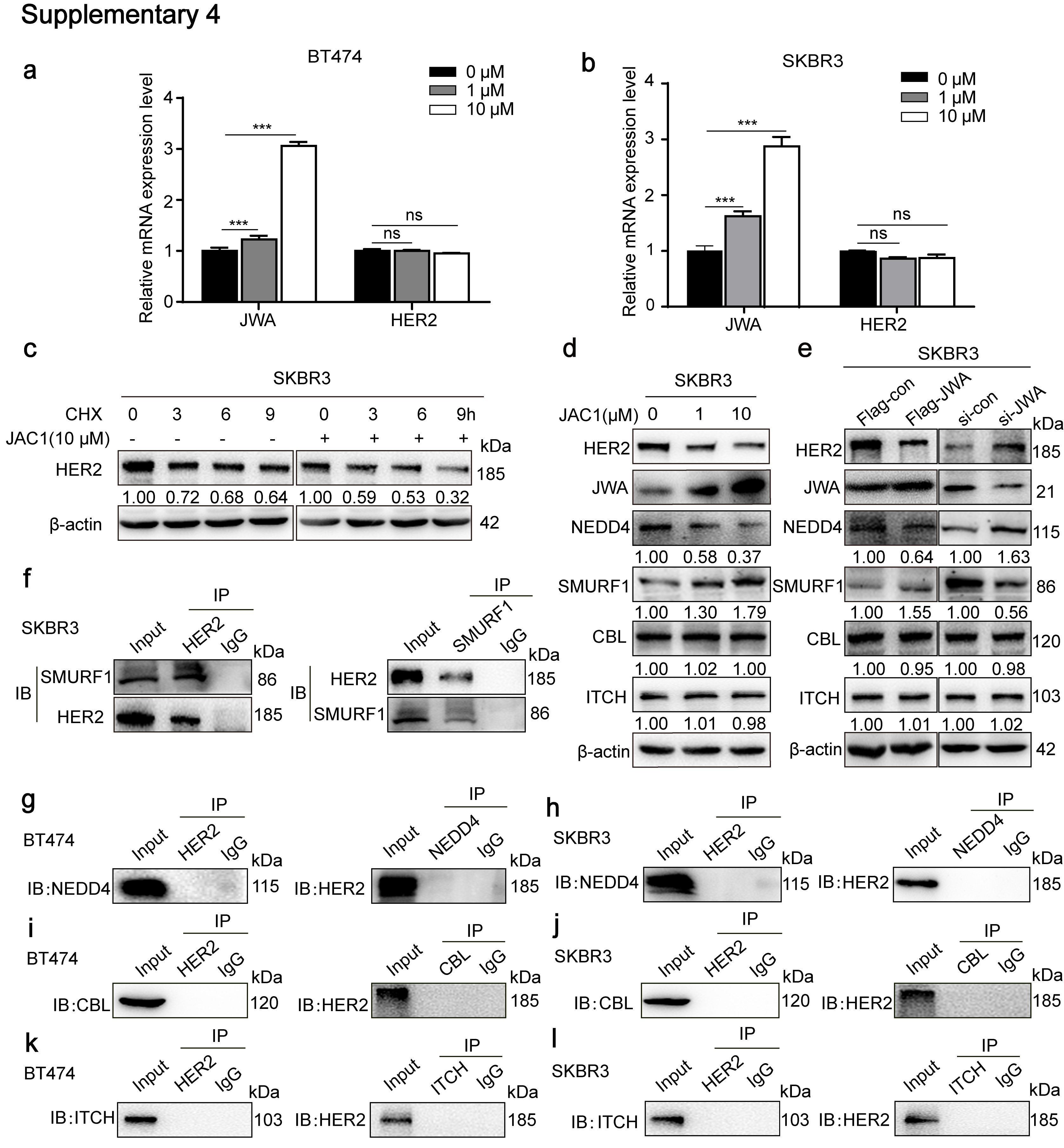

Supplement: Supplementary file 5 — Supplementary Figure 4 [file 41420_2021_426_MOESM5_ESM.tif]

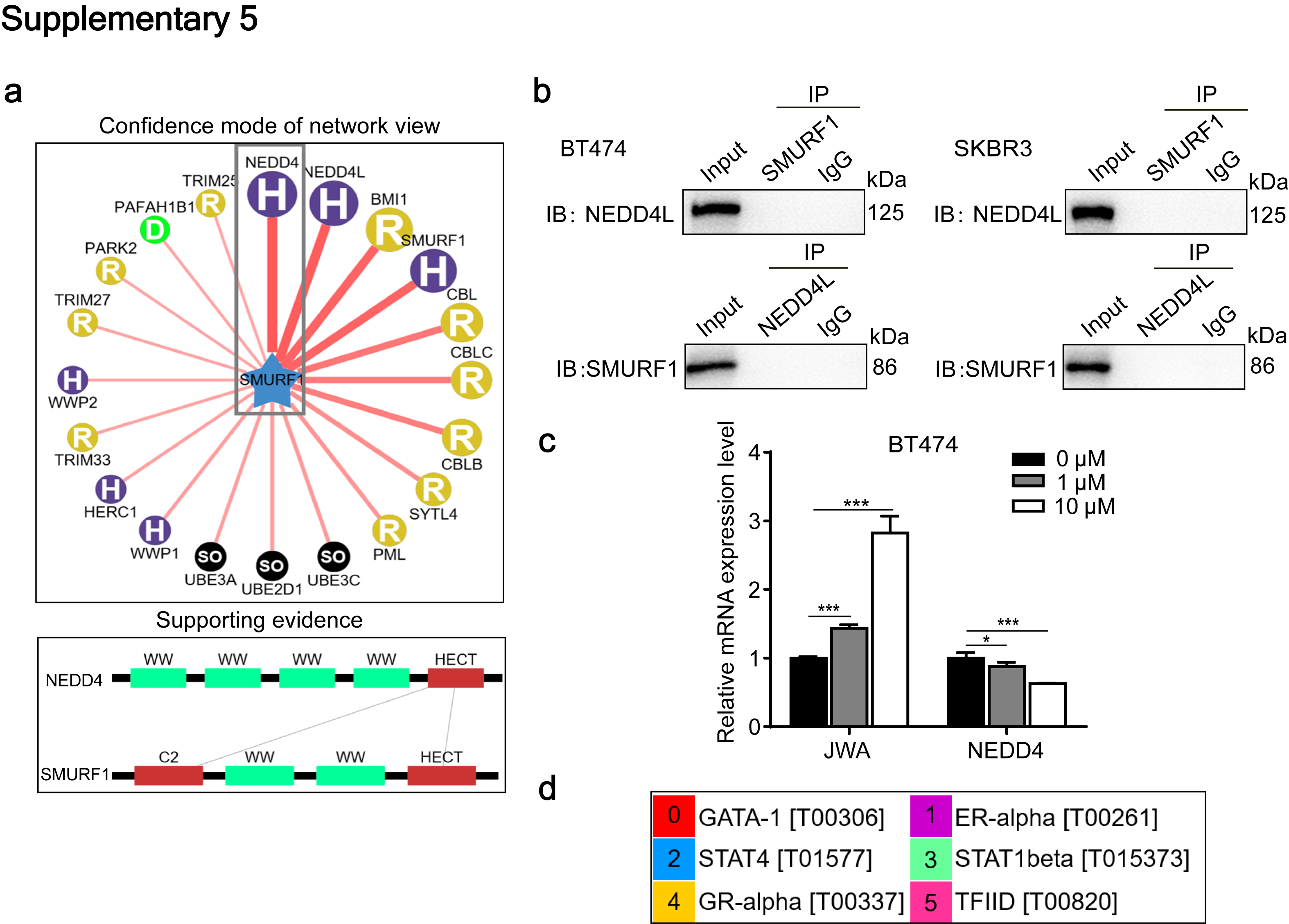

Supplement: Supplementary file 6 — Supplementary Figure 5 [file 41420_2021_426_MOESM6_ESM.tif]

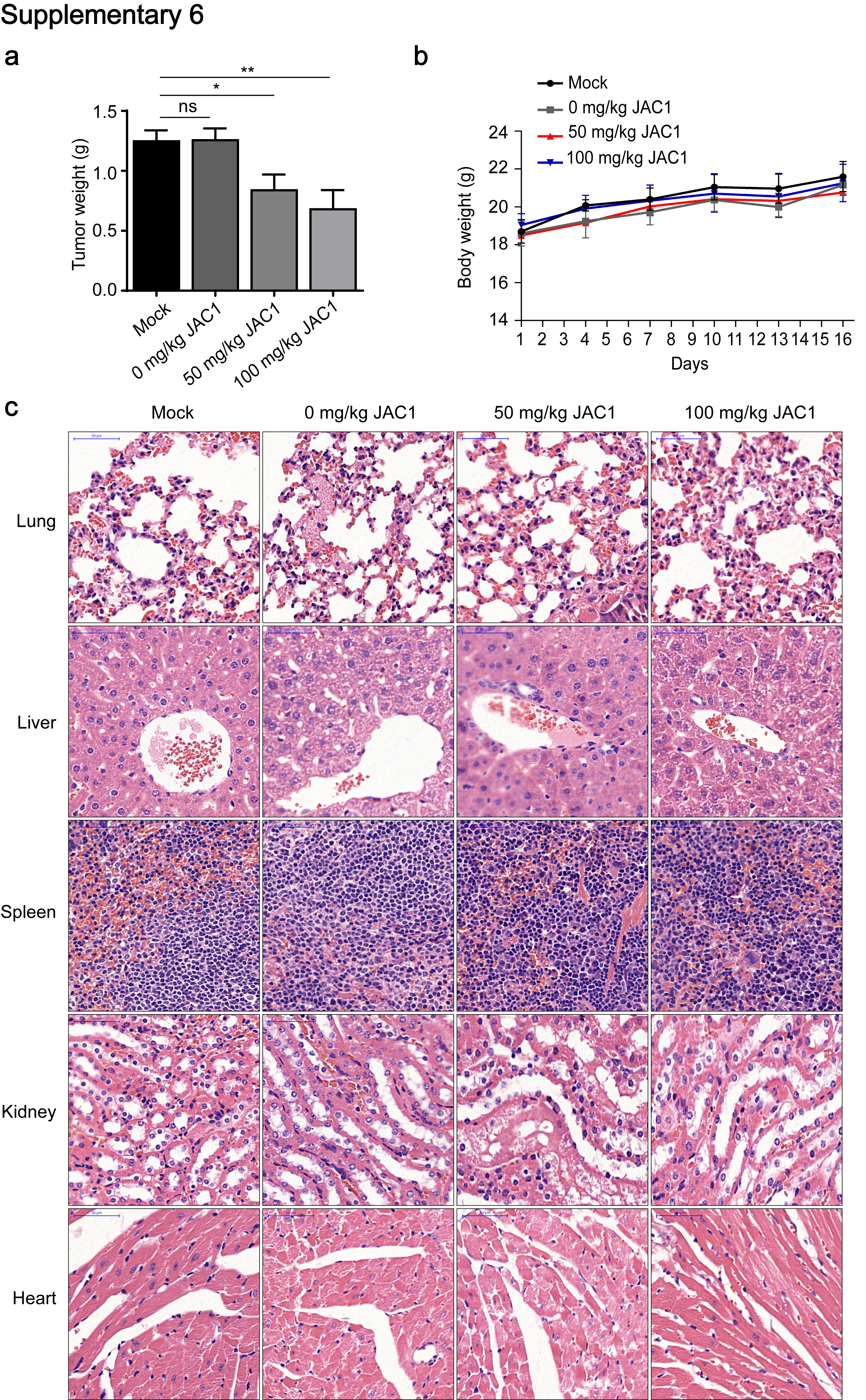

Supplement: Supplementary file 7 — Supplementary Figure 6 [file 41420_2021_426_MOESM7_ESM.tif]

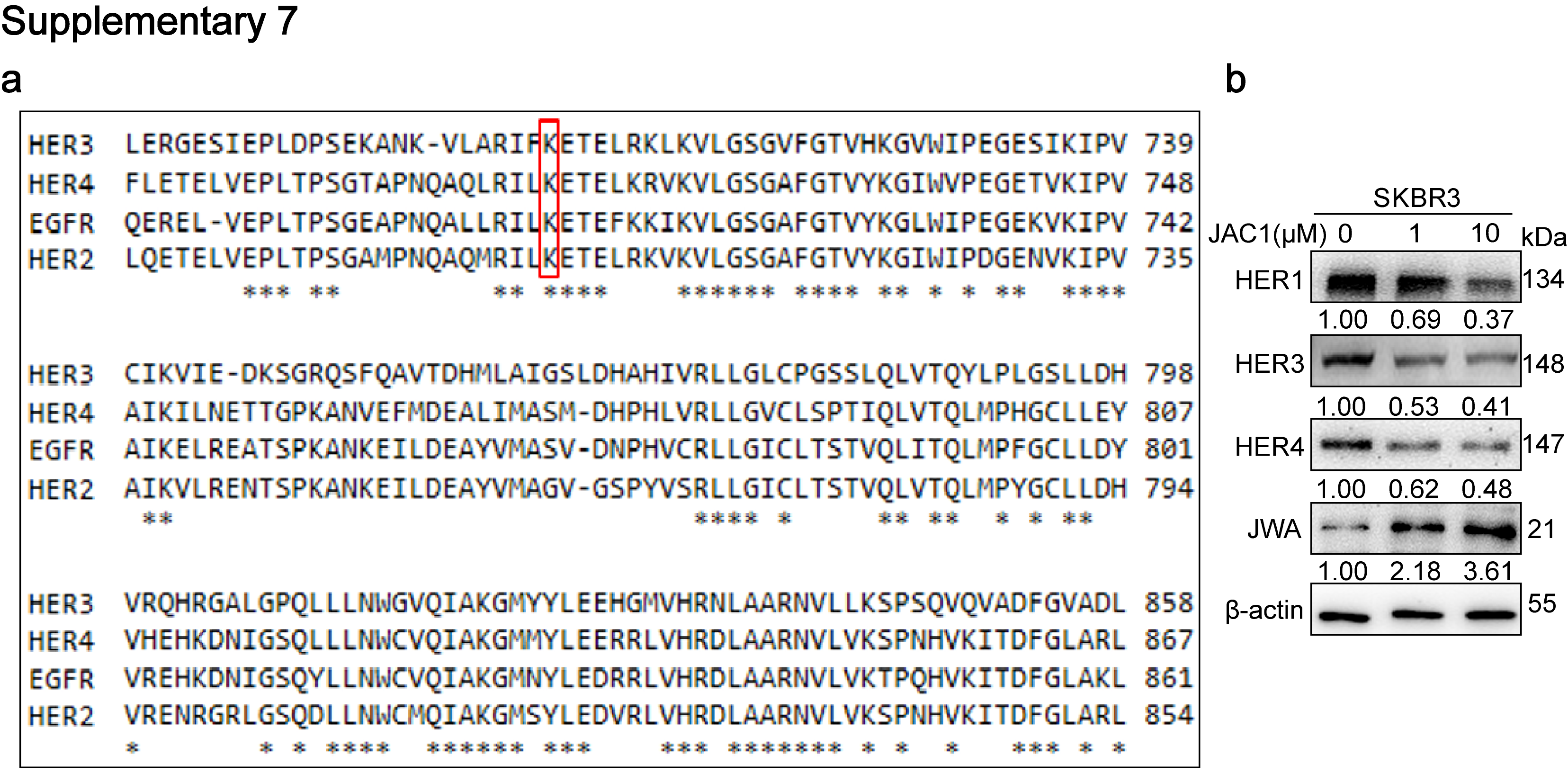

Supplement: Supplementary file 8 — Supplementary Figure 7 [file 41420_2021_426_MOESM8_ESM.tif]
